# Factors Associated with Implanon Discontinuation among Women of Reproductive Age in Ethiopia: A Systematic Review and Meta-Analysis

**DOI:** 10.1155/2022/9576080

**Published:** 2022-08-18

**Authors:** Zenebe Tefera, Mandefro Assefaw, Sindu Ayalew, Wondimnew Gashaw, Mengistu Abate, Kibir Temesgen, Nigusie Abebaw, Melaku Yalew

**Affiliations:** ^1^Department of Midwifery, College of Medicine and Health Science, Wollo University, Dessie, Ethiopia; ^2^Department of Epidemiology and Biostatistics, College of Medicine and Health Science, Wollo University, Dessie, Ethiopia

## Abstract

**Background:**

Implanon is a long-acting contraceptive method that is extremely effective in preventing pregnancy with a clinical failure rate of less than 1%. Despite these, the rate of Implanon discontinuation is a common problem in various societies and exposes women to unwanted conception and its consequences.

**Objective:**

The current study sought to find and consolidate relevant literature on Implanon discontinuation and associated factors in Ethiopia.

**Methods:**

Medline, PubMed, Cochrane Library, EMBASE, and Google Scholar databases were systematically searched for studies published in English before December 2021. The included studies were critically appraised using the JBI instrument for observational studies. STATA version 16 was used for analysis. The presence of statistical heterogeneity was checked using Cochran's *Q* test, and its level was quantified using *I*^2^ statistics. A pooled estimate of the proportion of outcome variables was calculated. To measure the effect size, pooled odds ratios with 95% CI were computed.

**Results:**

The pooled prevalence of Implanon discontinuation in Ethiopia was 32.89%, 95% CI: 24.11%, 41.66%. Experiencing side effects (OR = 2.52, 95% CI 1.75, 3.65), having no children (OR = 1.69, 95% CI 1.15, 2.47), not having received preinsertion counselling (OR = 1.65, 95% CI 1.36, 2.00), having no postinsertion appointment (OR = 2.97, 95% CI 2.10, 4.21), and not satisfied with the service (OR = 2.72, 95% CI 2.47, 5.59) were significantly associated with Implanon discontinuation.

**Conclusion:**

The pooled prevalence of Implanon discontinuation in Ethiopia was high. Experiencing side effects, having no child, not receiving preinsertion counselling, having no follow-up appointment, and not being satisfied with the service were significantly associated with Implanon discontinuation. Therefore, healthcare providers should offer preinsertion counselling in accordance with national family planning guidelines, emphasizing the method's advantages and side effects.

## 1. Introduction

Family planning is one of the most efficacious and cost-effective means of improving individual health, gender equity, family well-being, and national development [1]. It is a key component of basic health services that benefits the health and well-being of women, children, families, and communities [2]. Contraception plays an important role in advancing maternal and newborn health, reducing maternal mortality, and ensuring universal access to reproductive healthcare [3, 4]. It helps women to avoid unplanned and/or unwanted pregnancies and prevents unsafe abortions [5].

Human and financial resources are limited in less-developed regions of the world, particularly sub-Saharan Africa and South Asia, modern contraceptive use is low, the unmet need for modern contraception is high, and maternal morbidity and mortality are high as a result [1]. Globally, in 2019, 44 percent of women of reproductive age used modern methods of contraception. This comprises 91 percent of all contraceptive users; the remaining 9% use traditional methods [6]. Contraceptive use among women of reproductive age was higher than 55% in 37 countries in 2019 and lower than 20% in 23 countries [6].

The 2015 Costed Implementation Plan for Family Planning in Ethiopia, developed by the Ministry of Health, was aimed at increasing the contraceptive prevalence to 55% by 2020 [7]. However, the 2019 Ethiopian Demographic and Health Survey report shows that the contraceptive prevalence rate among currently married women aged 15-49 in Ethiopia is 41%, of which implants account for 9% [8]. To address this gap, since 2009, the government of Ethiopia has embarked on an Implanon scale-up initiative aimed at expanding access to and enhancing the use of Implanon at the community level by enabling health extension workers to provide Implanon insertion services [9, 10].

Contraceptive discontinuation is common in developing countries (18%–63%), and most of these discontinuations are among women who are still in need of contraception [10]. Implanon is a long-acting contraceptive method that is extremely effective in preventing pregnancy with a clinical failure rate of less than 1% [11]. Despite this, the rate of Implanon discontinuation before its due date is a common problem in various societies and exposes women to unwanted conception and its consequences [12].

In Ethiopia, the prevalence of Implanon discontinuation ranges from 16% to 65% [13, 14]. Studies in Ethiopia shows that factors such as not receiving preinsertion counselling, not being satisfied with the service, not having a child, experiencing the side effects of the method, and not having postinsertion appointment were associated with early Implanon discontinuation [13–20]. However, the findings of each study were highly variable. As a result, carefully summarizing all the available relevant evidence will help in decision-making to overcome the problem. In this systematic review and meta-analysis, we aimed to synthesize and evaluate the available studies on the prevalence and associated factors of Implanon discontinuation.

## 2. Materials and Methods

### 2.1. Protocol Registration and Review Reporting

The protocol of this systematic review and meta-analysis was registered at the International Prospective Register of Systematic Reviews (PROSPERO) with the registration number CRD42020193678. The review process followed the Preferred Reporting Items for Systematic Reviews and Meta-Analyses (PRISMA), which guides the reporting of systematic reviews and meta-analyses [21, 22].

### 2.2. Inclusion Criteria

Observational studies (cross-sectional, case-control, and cohort) published in English language were included. Peer-reviewed papers published before December 20, 2021, which were conducted in Ethiopia on Implanon discontinuation and associated factors among reproductive age women were included.

### 2.3. Exclusion Criteria

Conference abstracts and studies that were purely qualitative and had different methods of outcome measurement and studies that cannot be accessed in full after all possible options have been tried were excluded.

### 2.4. Search Strategy

We searched peer-reviewed studies in Medline, PubMed, Cochrane Library, EMBASE, and Google Scholar, using the following terms: “Implanon discontinuation OR early Implanon removal OR premature Implanon discontinuation” AND “associated factors OR determinants” AND “reproductive-age women” AND “Ethiopia.” The existence of similar systematic reviews and meta-analysis was checked to avoid duplication. The reference lists of the identified studies were reviewed to identify additional articles.

### 2.5. Study Selection

All retrieved studies were exported to the EndNote X8 reference manager software. Duplicate articles were identified and excluded. The titles, abstracts, and full texts of the identified studies were reviewed for screening and selected as needed. The PRISMA flow diagram was applied to summarize and synthesize the selection procedure and process of the articles [23].

### 2.6. Data Extraction and Quality Appraisal

The data extraction was performed by the two reviewers using an appropriate Microsoft Excel data extraction sheet, which was adapted based on a standardized JBI data extraction format. The pieces of information that were included in the extraction are primary author, study year, study period, publication year, region of the study, sample size, study design, study setting, prevalence of Implanon discontinuation, and odds ratio of determinants of Implanon discontinuation. Two reviewers (KT and NA) assessed the quality of the articles to reduce the risk of selection bias. The Joanna Briggs Institute (JBI) critical appraisal checklist was used to evaluate the quality of the studies [24].

### 2.7. Outcome Measurement

This study yielded two major findings. The primary finding was the magnitude of Implanon discontinuation, which was computed by dividing the total number of Implanon discontinuation cases by the total number of reproductive age women enrolled in the studies and multiplying by 100. The factors associated with the discontinuation of Implanon among women of reproductive age were the second outcome. Using binary outcomes from primary research, the odds ratio for the main variables was calculated.

### 2.8. Data Processing and Analysis

For further analysis, the extracted data was exported to Stata version 16 software. A meta-analysis was run using a random effects model because high heterogeneity was observed (*I*^2^ = 97.7%). The presence of heterogeneity among the studies was determined and quantified using Cochran's *Q* test and the *I*^2^ statistics, respectively. Heterogeneity analyses (like subgroup analysis, sensitivity analysis, and meta-regression) were conducted to assess the sources of heterogeneity. The possible risk of publication bias was examined by using an inspection of the funnel plot as well as Egger's and Begg's tests with a *p* value less than 0.05, which was used to declare the statistical significance of publication bias. The DerSimonian and Laird approach of the random effects model was used to calculate the pooled effect measure, which was given as an odds ratio with a respective 95 percent confidence interval.

## 3. Results

### 3.1. Study Selection

**Figure 1:**
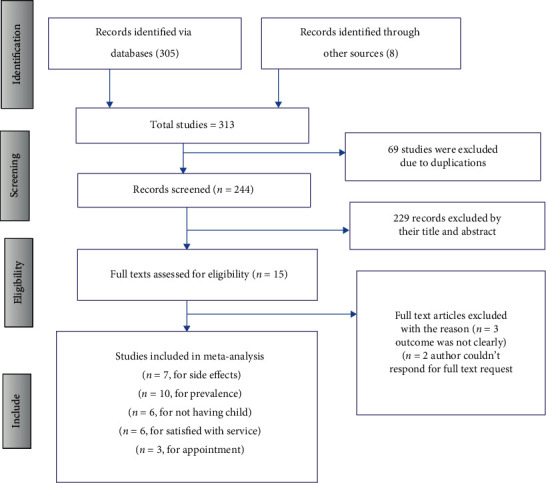
PRISMA flow diagram of the included studies for meta-analysis of prevalence of Implanon discontinuation among reproductive age women in Ethiopia, 2022.

A total of 313 articles were retrieved from databases and search engines. 244 articles were left when duplicate studies were removed. Then, the remaining articles were assessed by their titles and abstracts, and 229 studies were screened and excluded due to nonrelevance to the study. The remaining 15 full-text articles were evaluated for eligibility based on the inclusion and exclusion criteria. Finally, 10 articles are included in the systematic review and meta-analysis (Figure 1).

### 3.2. Study Characteristics

The 10 included studies were conducted between 2014 and 2021. Concerning their study, there are three in the Southern nation, nationalities, and Peoples (SNNP) region [15, 16, 20], four in the Amhara region [13, 18, 19, 25], two in Oromia [17, 26], and one in the Tigray region [14]. The sample size ranges from a minimum of 264 from the Tigray region [14] to a maximum of 711 from the Southern nation, nationalities, and Peoples (SNNP) region [15].

In a current meta-analysis, 4403 reproductive age women were included to estimate the pooled magnitude of Implanon discontinuation in Ethiopia. Eight studies selected in this review were cross-sectional in design, while two studies were case-control. Three studies were conducted at the institution level [13, 18, 27], and seven were community-based studies [14–17, 19, 20, 26]. Regarding the response rate, almost all studies had a good response rate (>90%) (Table 1).

### 3.3. The Prevalence of Implanon Discontinuation

**Figure 2:**
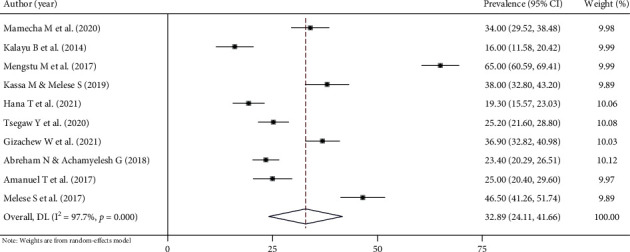
Forest plot for the pooled prevalence of Implanon discontinuation among reproductive age women, a systematic review and meta-analysis, Ethiopia, 2022.

**Figure 3:**
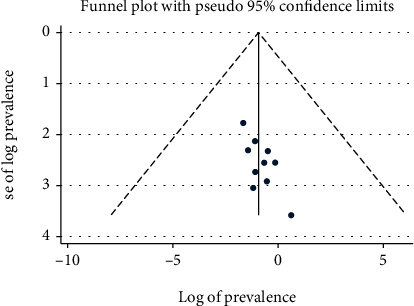
Funnel plot with 95% confidence limit of the pooled prevalence of Implanon discontinuation in Ethiopia, 2022.

This systematic review and meta-analysis shows that the pooled prevalence of Implanon discontinuation in Ethiopia was 32.89% (95% CI: 24.11%, 41.66%). High heterogeneity was observed across the included studies (*I*^2^ = 97.7%, *p* < 0.000), which is suggestive of using a random effects model (Figure 2). The potential sources of heterogeneity were assessed using sample size and year of publication, but none of these variables were found to be statistically significant. The funnel plot shows more or less symmetric distribution, which indicates the absence of publication bias (Figure 3). In addition, Egger's weighted and Begg's tests were computed to see the significant presence of publication bias. Both tests showed no statistically significant presence of publication bias (*p* = 0.1366 and *p* = 0.2105), respectively.

### 3.4. Subgroup Analysis

Subgroup analysis was done using study setting, study design, and region where the studies are conducted to assess potential variability between studies. The highest prevalence of Implanon discontinuation was observed in Amhara region (43.37%) (95% CI: 26.13%, 60.60%) and lowest prevalence in Tigray region (16%) (95% CI: 11.58%, 20.425) (Table 2).

### 3.5. Sensitivity Analysis

**Figure 4:**
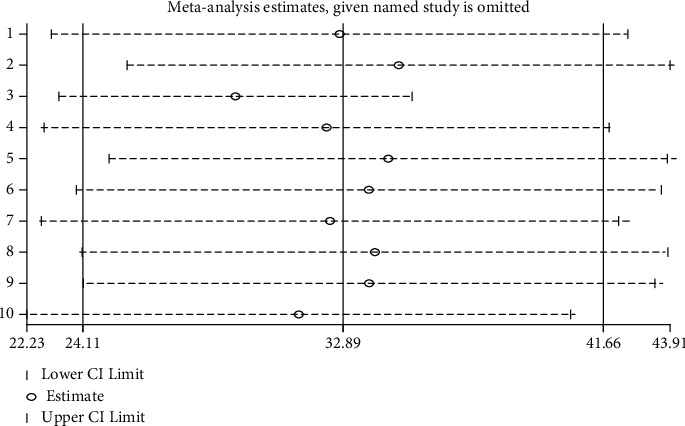
Sensitivity analysis for studies included in a systematic review and meta-analysis of Implanon discontinuation in Ethiopia, 2022.

We have conducted influential analysis to see the effect of individual study on the observed heterogeneity. However, the result showed that there was no strong evidence for the effect of a single study on the overall meta-analysis result (Figure 4).

### 3.6. Factors Associated with Implanon Discontinuation

#### 3.6.1. Association between Experiencing Side Effects and Implanon Discontinuation

**Figure 5:**
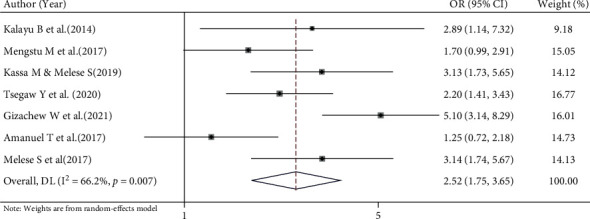
Forest plot for the pooled estimate of the effect of side effects on Implanon discontinuation, a systematic review and meta-analysis, Ethiopia, 2022.

This systematic review and meta-analysis uses a random effects model to estimates the pooled association between experiencing side effects of Implanon and its discontinuation using eight selected studies [13, 14, 16, 18]. The pooled finding showed that the odds of Implanon discontinuation were 2.52 times higher among women who had experienced side effects of Implanon as compared to women who had not experienced the side effects (OR = 2.52, 95% CI 1.75, 3.65) (Figure 5).

#### 3.6.2. Association between Had No Postinsertion Appointment and Implanon Discontinuation

**Figure 6:**
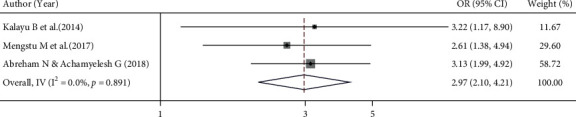
Forest plot for the pooled estimate of the effect of follow-up appointment on Implanon discontinuation, a systematic review and meta-analysis, Ethiopia, 2022.

In this meta-analysis, we examined the association between appointment for follow-up and Implanon discontinuation using three studies [13–15]. A fixed effects model was used to estimate the pooled association between appointments for follow-up and Implanon discontinuation (*I*^2^ = 0.0%, *p* value = 0.891). The pooled finding showed that the odds of Implanon discontinuation were 2.97 times higher among women who had no postinsertion appointments compared to women who had appointments after Implanon insertion (OR = 2.97, 95% CI 2.10, 4.21) (Figure 6).

#### 3.6.3. Association between Counselling and Implanon Discontinuation

**Figure 7:**
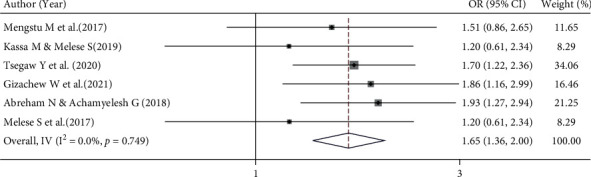
Forest plot for the pooled estimate of the effect of counselling on Implanon discontinuation, a systematic review and meta-analysis, Ethiopia, 2022.

This meta-analysis also examines the association between preinsertion counselling and Implanon discontinuation using six studies [13, 15, 18, 19, 25, 26]. A fixed effects model was used to estimate the pooled association between receiving preinsertion counselling and Implanon discontinuation (*I*^2^ = 0.0%, *p* value = 0.749). The pooled finding showed that the odds of Implanon discontinuation were 1.65 times higher among women who had no preinsertion counselling compared to women who had received preinsertion counselling (OR = 1.65, 95% CI 1.36, 2.00) (Figure 7).

#### 3.6.4. Association between Service Satisfaction and Implanon Discontinuation

**Figure 8:**
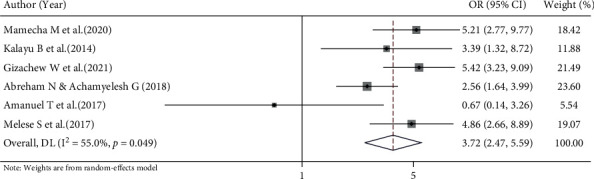
Forest plot for the effect of not being satisfied with service on Implanon discontinuation, a systematic review and meta-analysis, Ethiopia, 2022.

We have examined the association between service satisfaction and Implanon discontinuation using six articles [14–16, 19, 20, 25]. A random effects model was used to estimate the pooled association between service satisfaction and Implanon discontinuation (*I*^2^ = 55%, *p* value = 0.049). The result shows that the odds of Implanon discontinuation were 3.72 times higher among women who were poorly satisfied with the service provided as compared to their counterparts (OR = 3.72, 95% CI 2.47, 5.59 (Figure 8).

#### 3.6.5. Association between Not Having a Child and Implanon Discontinuation

**Figure 9:**
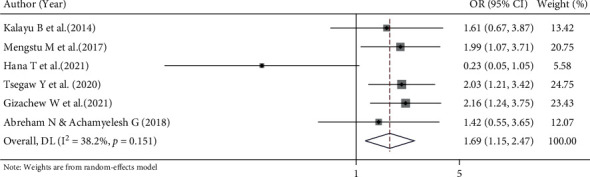
Forest plot for the pooled estimate of the effect of not having a child on Implanon discontinuation, a systematic review and meta-analysis in, Ethiopia, 2022.

This meta-analysis further examined the association between not having a child and Implanon discontinuation using six studies [13–15, 17, 18, 25]. A random effects model was used to estimate the pooled association between not having a child and Implanon discontinuation (*I*^2^ = 38.2%, *p* value = 0.151). The analysis shows that the odds of Implanon discontinuation were 1.69 times higher among women who had no children as compared to their counterparts (OR = 1.69, 95% CI 1.15, 2.47) (Figure 9).

## 4. Discussion

Globally, an estimated 830 women die of pregnancy-related causes each day, amounting to more than 300,000 deaths each year; 99% of these deaths occur in low- and middle-income countries [29]. However, access to family planning can greatly reduce these mortality rates. Providing access to contraception for all those who need it would reduce worldwide maternal deaths by 29% [30]. Family planning can reduce maternal mortality by preventing unwanted pregnancy and unsafe abortion and by promoting healthy pregnancies [31]. One of the most efficient and secure family planning methods is the Implanon, which contains the hormone etonogestrel, which has been shown to reduce dysmenorrhea and dyspareunia [32]. Evidence suggests that etonogestrel can help patients with ovarian cysts that are thought to be of endometriotic origin feel less pain in their pelvis and have improved sexual function and quality of life [32]. Insertion of the contraceptive Implanon at an alternative subdermal scapular site has been reported in the literature, which will reduce the complications that occur at the insertion site and will make it safer to use. This insertion site represents an ideal alternative location for the contraceptive Implanon due to its location far from danger zones of neurovascular structures, inaccessibility to patients with mental illnesses, and underlying bony structures preventing unintentional deep insertion [32, 33]. This will enhance the utilization of contraceptive Implanon.

Despite global advances in access to family planning services, 218 million women in low- and middle-income countries still experience an unmet need for modern contraception [34], yet 12% of women in most regions of the world have an unmet need for family planning [34]. Contraception discontinuation by women of reproductive age while still in need is a serious public health concern [35]. Contraceptive discontinuation is an important determinant of contraceptive prevalence, as well as unwanted fertility and induced abortion [36]. Implanon is one of the long-acting contraceptive method that is highly tolerable and extremely effective at preventing pregnancy with a clinical failure rate of less than 1% [11]. Despite good tolerance and effectiveness, many women discontinue Implanon use before its due date [14].

Previous studies in Ethiopia reported that a significant proportion of mothers discontinue Implanon use before the completion of three years [13–20, 34, 35]. The discontinuation rate varies from study to study, but there is no estimate made based on reviewing available research in Ethiopia that could estimate the overall rate of Implanon discontinuation. Therefore, this study evaluated 10 studies [13–20, 25, 26] and estimated the pooled discontinuation rate of Implanon. In this study, we have conducted a meta-analysis of eight cross-sectional and two case-control studies, including 4403 reproductive age women, derived from 32.89%, 95% CI = 24.11%, and 41.66%, a national pooled estimate of Implanon discontinuation. The prevalence of Implanon discontinuation was high in the Amhara region, 65% (95% CI 60.59, 69.41) [13], and low in the Tigray region, 16 (95% CI = 11.58% 20.42%) [14]. This finding is in line with a study conducted in India, in which the prevalence of Implanon discontinuation due to different reasons was 30.5% [37]. This study's finding, however, was higher than the previous national survey study conducted by the Federal Ministry of Health (Ethiopian) and Family Health International (FHI 360), in which 17% of the women reported removing their Implanon before the recommended three-year postinsertion removal date [38]. The discrepancy could be ascribed to the difference in study design and sample size where the 1860 participants were included in the previous study while 4632 participants included in this study.

According to this review, experiencing the side effects, not being satisfied with the service provided, not receiving preinsertion counselling, not having a postinsertion follow-up appointment, and having no live child were factors significantly associated with Implanon discontinuation in Ethiopia. Our pooled results suggested that women who had experienced the side effects were 2.52 times more likely to discontinue the use of Implanon before its due date for removal. This finding is supported by studies conducted in Buffalo City Metropolitan Municipality and KwaZulu-Natal, South Africa [33, 39]. A study conducted in the four regions of Senegal shows that the most frequently cited reason for discontinuation among implant users was a dislike of side effects [40]. Further analysis of 2017 Demographic and Health Survey in Indonesia strengthens our finding that discontinuations of the method due to side effects are by far the largest contributor to the overall reasons for the discontinuation of the method in Indonesia [41].

This meta-analysis also suggests that the likelihood of Implanon discontinuation among women of reproductive age who were not satisfied by the service provided was 3.72 times higher than its counterpart. Likewise, women who had no postinsertion follow-up appointment were 2.97 times more likely to discontinue the method compared to those who had a follow-up appointment (OR = 2.97, 95% CI 2.10, 4.21). However, as indicated in a study conducted in Atlanta, USA, it was difficult to determine what effect, if any, follow-up visits or contacts had on method continuation or correct use [42]. The difference could be attributed to defence in the study setting and study period.

Based on this review, women who had not received preinsertion counselling were 1.65 times more likely to discontinue Implanon use before its due date compared to women who had received preinsertion counselling (OR = 1.65, 95% CI 1.36, 2.00). Receiving information on the advantages and side effects of the method is one of the fundamental elements of quality of care, which will enhance the continued use of the method [43]. In this study, women who had no children were 1.69 times more likely to discontinue the method compared to those who had children (OR = 1.69, 95% CI 1.15, 2.47). This could be due to women who have never had children having a greater desire for pregnancy. As a result, the women will discontinue the method.

This review has several strengths, including the following: this review tried to identify all potential determinants of Implanon discontinuation. Moreover, we used comprehensive search strategies and the PRISMA checklist to improve the quality of the review. The protocol for this manuscript was registered on PROSPERO, whereas this review has limitations, such as the review included studies that were published only in the English language.

## 5. Conclusions

This systematic review and meta-analysis study reveals that the national pooled prevalence of Implanon discontinuation is high as compared to other studies. Experiencing the side effects, not being satisfied with the service provided, not receiving preinsertion counselling, having no postinsertion follow-up appointment, and not having a live child were found to be determinant factors for Implanon discontinuation in Ethiopia. Therefore, healthcare providers should offer preinsertion counselling in accordance with national family planning guidelines, emphasizing the method's advantages and side effects. Implanon retention could be boosted if appointment follow-up services were improved. Working to improve customers' perceived satisfaction could help to reduce Implanon discontinuation.

## Figures and Tables

**Table 1:** Characteristics of the selected studies for systematic review and meta-analysis of the factors associated with Implanon discontinuation in Ethiopia, 2022.

ID	Author (publication year)	Study year	Region	Study design	Study setting	Sample size	Prevalence	Quality score
1	Mesha et al. (2020) [20]	2018	SNNP	Cross-sectional	Community	430	34%	7
2	Birhane et al. (2015) [14]	2014	Tigray	Cross-sectional	Community	264	16%	7
3	Melkamu Asaye et al. (2017) [13]	2016	Amhara	Cross-sectional	Institutional	449	65%	8
4	Dagnew et al. (2019) [25]	2018	Oromia	Cross-sectional	Community	335	38%	7
5	Tesfaye et al. (2021) [17]	2020	Oromia	Cross-sectional	Community	430	19.3%	8
6	Yehuala et al. (2020) [18]	2019	Amhara	Case-control	Institution	559	25.2%	8
7	Gizachew et al. (2021) [28]	2017	Amhara	Cross-sectional	Institution	537	36.9%	7
8	Nageso and Gebretsadik [15]	2017	SNNP	Cross-sectional	Community	711	23.4%	7
9	Tadesse et al. (2017) [16]	2017	SNNP	Case-control	Community	340	25%	6
10	Siyoum et al. (2017) [19]	2016	Amhara	Cross-sectional	Community	348	46.5%	5

**Table 2:** Subgroup analysis for the prevalence of Implanon discontinuation among reproductive age women in Ethiopia (*n* = 10).

Variables	Included studies	Sample size	Prevalence
By study setting			
Community based	7	2858	28.77 (21.39, 36.14)
Institutional based	3	1545	42.34 (19.70, 64.98)
By region			
Amhara	4	1893	43.37 (26.13, 60.60)
SNNP	3	1481	27.36 (20.98, 33.73)
Tigray	1	264	16.0 (11.58, 20.42)
Oromia	2	765	28.56 (10.23, 46.88)
Overall	10	4403	32.89 (24.11, 41.66)

## Data Availability

All the data are available from the corresponding author upon reasonable request.
